# Supplementary material for: Ipomoeassin-F inhibits the in vitro biogenesis of the SARS-CoV-2 spike protein and its host cell membrane receptor
Source: J Cell Sci. 2021 Feb 19;134(4):jcs257758. doi: 10.1242/jcs.257758 (PMC7904091; doi:10.1242/jcs.257758)
Supplement: Supplementary information [file joces-134-257758-s1.pdf]

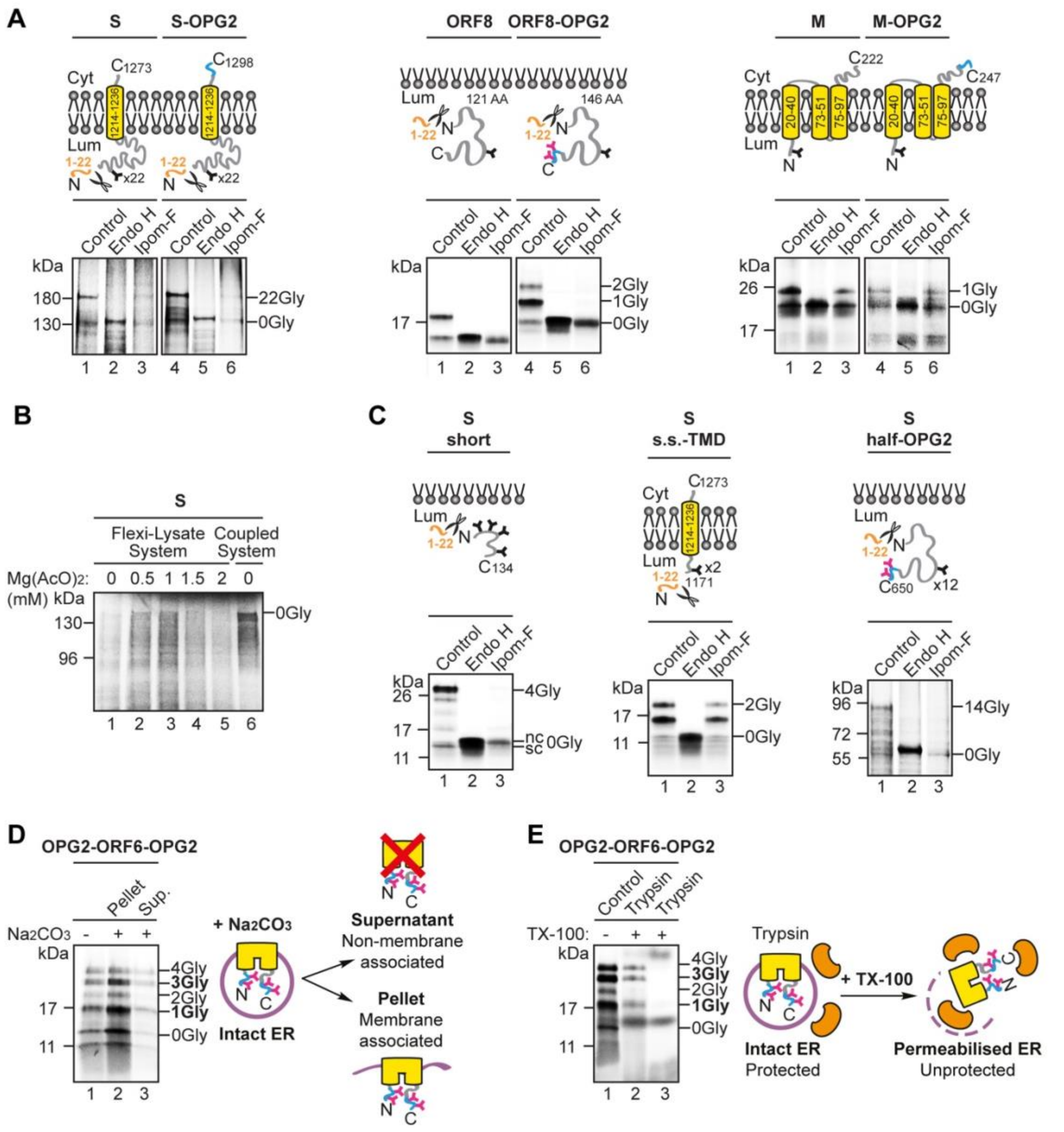

Fig. S1. Additional studies using ER microsomes, Related to Figures 2 and 3.
(**A**) Non-tagged (lanes 1-3) and OPG2-tagged (lanes 4-6) versions of the SARS-CoV-2 spike protein (S, S-OPG2), ORF8 (ORF8, ORF8-OPG2) and membrane protein (M, M-OPG2) were synthesised in rabbit reticulocyte lysate supplemented with ER-derived canine pancreatic microsomes in the absence and presence of Ipom-F (lanes 1 and 3). Phosphorimages of membrane-associated products resolved by SDS-PAGE together with representative substrate outlines are shown. N-glycosylated (X-Gly) versus non-N-glycosylated (0Gly) species were identified by treatment with endoglycosidase H (Endo H, lanes 2 and 5). (**B**) The S protein was synthesised in a Flexi® rabbit reticulocyte system with varying concentrations of magnesium acetate (lanes 1-5) and a TNT® Coupled system (lane 6) in the absence of ER-derived

microsomes. 5% of the total reaction material was resolved by SDS-PAGE and visualised by phosphorimaging. (**C**) The ER import of truncated variants of the S protein (S-short, S-s.s.-TMD, S-half-OPG2) was analysed as described for **(A)**. (**D**) The membrane-associated products of the doubly tagged form of ORF6 (OPG2-ORF6-OPG2) were synthesised as in **(A)** and, following treatment with sodium carbonate buffer and centrifugation, the pellet, enriched for membrane-integrated material, and supernatant, largely containing peripherally membrane-associated material, were analysed for OPG2-ORF6-OPG2. (**E**) The membrane-associated products of OPG2-ORF6-OPG2 were treated with trypsin in the absence or presence of Triton-100 (TX-100, lanes 2-3).

Journal of Cell Science • Supplementary information

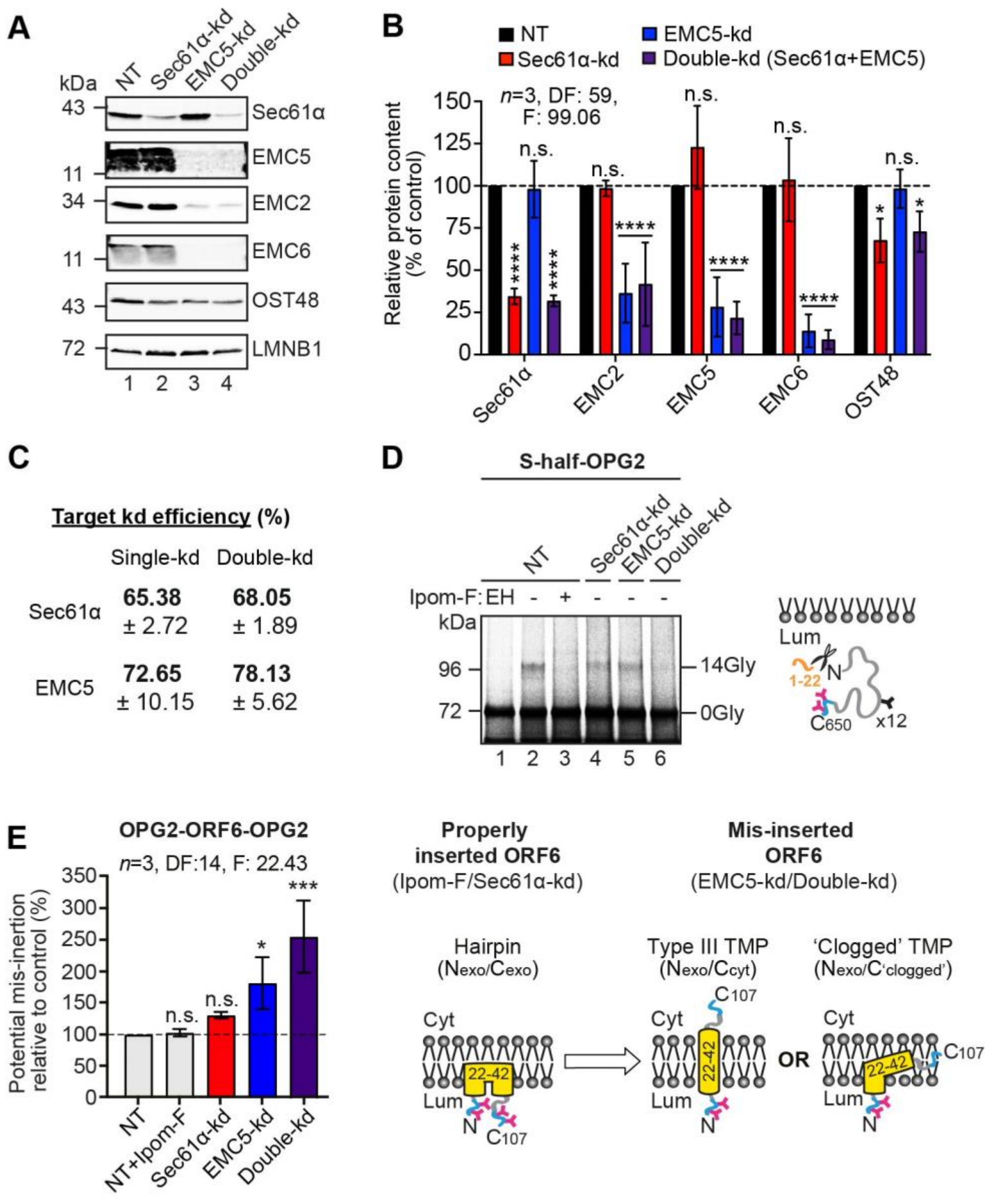

Fig. S2. Validation of Sec61 and/or EMC subunit depletions in SP cells, Related to Figure 3.

(A) The effects of transfecting HeLa cells with non-targeting (NT; lane 1), Sec61α-targeting (lane 2), EMC5-targeting (lane 3) and Sec61α+EMC5-targeting (lane 4) siRNAs were determined after semi-permeabilisation by immunoblotting for target genes (Sec61α, EMC5). Controls to assess destabilisation of the wider EMC complex (EMC2 and EMC6), any effect on the N-glycosylation machinery (the ER-resident 48 kDa subunit of the oligosaccharyl-transferase complex (OST48) and the quantity of SP cells used in each experiment (the nuclear protein Lamin-B1 (LMNB1)), are also shown. (**B**) The efficiencies of siRNA-mediated knockdown (bold) were calculated as a proportion of the signal intensity obtained with the NT control (set as 100%).

Quantitations are given as mean±s.e.m for three separate siRNA treatments (*n*=3) with statistical significance of siRNA-mediated knockdowns (two-way ANOVA, DF and F values shown in the figure) determined using Dunnett's multiple comparisons test. Statistical significance is given as n.s., non-significant >0.1; *, P < 0.05; ****, P < 0.0001. (**C**) Knockdown efficiencies (mean±s.e.m) for each of the target genes. (**D**) A truncated variant of the S protein (S-half-OPG2) was synthesised in rabbit reticulocyte lysate supplemented with SP cells with impaired Sec61 complex and/or EMC function and recovered by immunoprecipitation via the OPG2 tag. Radiolabelled products resolved by SDS-PAGE and analysed by phosphorimaging. N-glycosylated (14-Gly) versus non-N-glycosylated (0Gly) species were identified by treatment with endoglycosidase H (Endo H, lane 1). (**E**) Further analysis of the data presented in Fig. 3E of the main text. Here, the ratio of 3Gly and 4Gly bearing OPG2-ORF6-OPG2 N-glycosylated species relative to the 1Gly species present in the same sample was used as a proxy to estimate potential mis-insertion of the ORF6 protein in SP cells with impaired Sec61 complex and/or EMC function relative to the NT control (set to 100% efficiency). Quantitations are given as mean±s.e.m for independent translation reactions from separate siRNA treatments performed in triplicate (*n*=3) and statistical significance (two-way ANOVA, DF and F values shown in the figure) was determined using Dunnett's multiple comparisons test. Statistical significance is given as n.s., non-significant >0.1; *, P < 0.05; ***, P < 0.001.
