# Supplementary material for: Ipomoeassin-F inhibits the in vitro biogenesis of the SARS-CoV-2 spike protein and its host cell membrane receptor
Source: J Cell Sci. 2021 Feb 19;134(4):jcs257758. doi: 10.1242/jcs.257758 (PMC7904091; doi:10.1242/jcs.257758)
Supplement: Reviewer comments [file joces-134-257758_review_history.pdf]

Ipomoeassin-F inhibits the *in vitro* biogenesis of the SARS-CoV-2 spike protein and its host cell membrane receptor

Sarah O'Keefe, Peristera Roboti, Kwabena B. Duah, Guanghui Zong, Hayden Schneider, Wei Q. Shi and Stephen High
DOI: 10.1242/jcs.257758

Editor: David Stephens

Review timeline
Original submission: 30 November 2020
Editorial decision: 15 December 2020
First revision received: 21 December 2020
Accepted: 4 January 2021

Original submission

First decision letter

MS ID#: JOCES/2020/257758

MS TITLE: Ipomoeassin-F inhibits the in vitro biogenesis of the SARS-CoV-2 spike protein and its host cell membrane receptor

AUTHORS: Sarah O'Keefe, Peristera Roboti, Kwabena B Duah, Guanghui Zong, Hayden Schneider, Wei Q Shi, and Stephen High
ARTICLE TYPE: Short Report

We have now reached a decision on the above manuscript.

To see the reviewers' reports and a copy of this decision letter, please go to: https://submit-jcs.biologists.org and click on the 'Manuscripts with Decisions' queue in the Author Area. (Corresponding author only has access to reviews.)

As you will see, the reviewers gave favourable reports but raised some critical points that will require amendments to your manuscript. I hope that you will be able to carry these out, because I would like to be able to accept your paper.

There are a few minor comments to attend to and I would add to that a suggestion that you comment on the likely limitations of using current Sec61 inhibitors as antiviral drugs or of inhibiting ACE2 biogenesis. While I appreciate the reviewers comments on the limits to the degree of advance and understanding that you provide with this work, and having consulted on this, I can see that the work presents a timely and interesting piece of work that would be of interest to both cell biology and virology communities.

We are aware that you may be experiencing disruption to the normal running of your lab that makes experimental revisions challenging. If it would be helpful, we encourage you to contact us to discuss your revision in greater detail. Please send us a point-by-point response indicating where you are able to address concerns raised (either experimentally or by changes to the text) and where you will not be able to do so within the normal timeframe of a revision. We will then provide further guidance. Please also note that we are happy to extend revision timeframes as necessary.

© 2021. Published by The Company of Biologists under the terms of the Creative Commons Attribution License (https://creativecommons.org/licenses/by/4.0/).

Please ensure that you clearly highlight all changes made in the revised manuscript. Please avoid using 'Tracked changes' in Word files as these are lost in PDF conversion.

I should be grateful if you would also provide a point-by-point response detailing how you have dealt with the points raised by the reviewers in the 'Response to Reviewers' box. Please attend to all of the reviewers' comments. If you do not agree with any of their criticisms or suggestions please explain clearly why this is so.

Reviewer 1

Advance summary and potential significance to field

In this manuscript O'Keefe and colleagues use an in vitro translation/ translocation assay to evaluate the impact of ipomoeassin-F (ipom-F) on the entry of SARS-CoV2 (and host) proteins in the ER.

This a very simple/sober and nicely conducted study that documents how viral proteins enter the secretory pathway and how this process is dependent on sec61. The manuscript is well written and the data are robust.

This study provides experimental evidences for the potential use of sec61-mediated translocation inhibitors in antiviral therapeutic strategies.

Comments for the author

In its current state the manuscript would benefit from 3 minor additions to be addressed by the authors 1) in the text on p5, it could be stated in one additional sentence that for some proteins (e.g. S) the OPG2 tag is exclusively dedicated for immunoprecipitation (present in the cytosol) and not for Nglycan addition.
2) On p6 the authors conclude that M is not affected by ipom-F, but based on the results presented in Fig2B (M-OPG2) it appears that there is a reduction in band inntensity compared to control. What would be the explanation for this observation?
3) It would be a real asset for the manuscript if the authors could carry the same experiments as presented in fig3 on the SARS-CoV2 M protein. How would the combination of sec61 and EMC5 kd impact on the membrane insertion of this protein?

Reviewer 2

Advance summary and potential significance to field

In this manuscript the authors describe the effects of the Sec61 channel inhibitor Ipomoeassin-F on biogenesis of SARS-CoV-2 encoded proteins and on the ACE2 host cell receptor for the virus. The authors find that membrane integration of proteins with a type III topology is resistant to Ipomoeassin-F, whereas Sec61-mediated translocation or integration of secretory and type I proteins including ACE2 is sensitive to the drug. In addition, the virus ORF6 encodes an unusual hairpin protein whose biogenesis is also resistant to Ipomoeassin-F. Using knock down experiments the authors also explore the relative contributions of Sec61 and the EMC to biogenesis of the viral proteins. Based on their results the authors propose ER translocation inhibitors as potential anti-viral agents.

Comments for the author

The paper is technically very good, is well written with excellent figures, and the results fully support the authors' conclusions. I only have a few minor queries:
- typographical errors: line 421 Wellcome Trust, line 292 Zimmermann
- noun that goes with 'to quantify' is 'quantitation', not 'quantification (lines 168, 280, 392, legend Fig S3)

© 2021. Published by The Company of Biologists under the terms of the Creative Commons Attribution License (https://creativecommons.org/licenses/by/4.0/).

- p15, line 359: pH of the carbonate extraction buffer?
- line 364: why does the IP buffer contain methionine?
- lines 371-73: rationale for using EndoHf for small proteins, EndoH for large proteins?
- lines 339, 348: what is the concentration of the microsome stock? The authors only give percentage of the assay volume.

The paper does not, however, provide any mechanistic insight into the functions of Sec61 or EMC, or the inhibitory mechanism by Ipom-F beyond the results already described in the accompanying paper, with the exception of the biogenesis of the unusual lumenal viral hairpin protein. The second paper also shows that type III proteins are resistant to Ipom-F and that Sec61 and the EMC can cooperate to varying degrees in the biogenesis of membrane proteins. For a cell biology readership there is no intrinsic gain in information to have this confirmed with viral proteins. This manuscript is therefore better suited for a more specialized virology or infectious disease journal.

The proposal to use Sec61 inhibitors as antiviral drugs seems unrealistic in light of the extremely unpleasant and difficult to treat effects of the bacterial Sec61 inhibitor mycolactone in human patients. Also, given that ACE2 plays a role in blood pressure and blood flow regulation, both of which are risk factors in Covid-19, it would seem extremely hazardous to use an ACE2 biogenesis inhibitor to combat this infection.

First revision

Author response to reviewers' comments

Ipomoeassin-F inhibits the in vitro biogenesis of the SARS-CoV-2 spike protein and its host cell membrane receptor

O'Keefe, S et al (2020).

We thank the two reviewers for their thoughtful and constructive comments regarding our original submission and provide a detailed response to each of their points below.

General points

Based on the suggestion of the editor and reviewer 2, we have additionally commented on the likely limitations of using current Sec61 inhibitors as antiviral drugs, particularly since ACE2 biogenesis is also inhibited (See yellow highlighted text on pages 8 and 9).

To comply with formatting requirements, we have now included a running title, moved figure legends to the end of the document, reformatted how statistics are reported in figure legends (now mean±s.e.m), reformatted an article reference that has now been published and re-grouped our supplementary information into a separate file.

Reviewer 1:

In its current state the manuscript would benefit from 3 minor additions to be addressed by the authors

R1, point 1) in the text on p5, it could be stated in one additional sentence that for some proteins (e.g. S) the OPG2 tag is exclusively dedicated for immunoprecipitation (present in the cytosol) and not for N-glycan addition.

Response: We have included two additional sentences to make this point clear, see yellow highlighted text on page 4 of the revised manuscript.

R1, point 2i) On p6 the authors conclude that M is not affected by ipom-F, but based on the results

© 2021. Published by The Company of Biologists under the terms of the Creative Commons Attribution License (https://creativecommons.org/licenses/by/4.0/).

presented in Fig2B (M-OPG2) it appears that there is a reduction in band intensity compared to control.

Response: Our assay for membrane insertion is based on the ratio of N- glycosylated to non-glycosylated species and in the case of the M protein this is not altered. However, we agree that there is a loss of total signal for the M protein that is observed with both its non-tagged and OPG-tagged forms (see Fig. 2 and S1A).
Hence, our original conclusion that the M protein was unaffected is an oversimplification. We have now highlighted this consistently observed signal reduction in the main text. See yellow highlighted text on page 5 of the revised manuscript.

R1, point 2ii) What would be the explanation for this observation?

Response: On the basis of the limited data that is currently available, we know that the Sec61 inhibitor mycolactone has little if any effect on the levels of endogenous type III multi-pass TMPs (Morel et al. 2018). This is most likely because their first TMDs employ the EMC for membrane insertion as we show here for the type III E- protein (Figure 3). In this context, the effects of Ipom-F on the membrane insertion of subsequent TMDs that lie C-terminal of one that employs the EMC remains an open question that we intend to address in a future study (see also response to question 3).

R1, point 3) It would be a real asset for the manuscript if the authors could carry the same experiments as presented in fig3 on the SARS-CoV2 M protein. How would the combination of sec61 and EMC5 kd impact on the membrane insertion of this protein?

Response: This is clearly an important question, but we believe that it lies beyond the scope of our present study and respectfully conclude that it will be best answered as part of a future study of diverse multi-pass TMPs.

<u>Reviewer 2</u>

I only have a few minor queries:

R2, point 1) typographical errors: line 421 Wellcome Trust, line 292 Zimmermann

Response: Thank you for pointing out these typographical errors, they have now been corrected. See yellow highlighted text on pages 10 and 14 of the revised manuscript and in the supplementary information.

R2, point 2) noun that goes with 'to quantify' is 'quantitation', not 'quantification (lines 168, 280, 392, legend Fig S3)

Response: Thank you for flagging this grammatical error, the wording has been corrected. See yellow highlighted text on pages 13 and 21 of the revised manuscript and supplementary information.

R2, point 3) p15, line 359: pH of the carbonate extraction buffer?

Response: The pH is 11.3 and has been added to the methods section. See yellow highlighted text on page 12 of the revised manuscript.

R2, point 4) line 364: why does the IP buffer contain methionine?

Response: This is to prevent background from radiolabelled methionine. This is now mentioned in the text, see yellow highlighted text on page 12.

R2, point 5) lines 371-73: rationale for using EndoHf for small proteins, EndoH for large proteins?

Response: This is so that the unlabelled Endo H enzyme does not affect the migration of the

© 2021. Published by The Company of Biologists under the terms of the Creative Commons Attribution License (https://creativecommons.org/licenses/by/4.0/).

radiolabelled product. Depending on the molecular weight of the radiolabelled protein of interest, we use the form of EndoH that will not co-migrate with the translation products of interest. See yellow highlighted text on page 12 of the revised manuscript.

R2, point 6) lines 339, 348: what is the concentration of the microsome stock? The authors only give percentage of the assay volume.

Response: In fact, we state the concentration of the stock solution of nuclease- treated canine pancreatic microsomes that were used is OD280 = 44/mL a few lines above. See green highlighted text on page 11 of the revised manuscript.

R2, comment 1: This manuscript is therefore better suited for a more specialized virology or infectious disease journal.

Response: We have published two previous studies of the Sec61 inhibitor mycolactone in the Journal of Cell Science (McKenna et al 2016. J. Cell Sci. **129**: 1404-1415; McKenna et al 2017. J. Cell Sci. **130**: 1307-1320) and are delighted that the Editor handling our current manuscript considers that it “presents a timely and interesting piece of work that would be of interest to both cell biology and virology communities”.

R2, comment 2: The proposal to use Sec61 inhibitors as antiviral drugs seems unrealistic in light of the extremely unpleasant and difficult to treat effects of the bacterial Sec61 inhibitor mycolactone in human patients. Also, given that ACE2 plays a role in blood pressure and blood flow regulation, both of which are risk factors in Covid-19, it would seem extremely hazardous to use an ACE2 biogenesis inhibitor to combat this infection.

Response: This is of course a fair comment, and as with any small molecule inhibitor that targets a fundamental cellular pathway there are obvious caveats regarding their future use. On the other hand, it has been shown that the general translation inhibitor cycloheximide can significantly inhibit SARS-CoV-2 replication at concentrations that are not toxic to human cells in culture (**Bojkova et al. 2020**, as cited in manuscript). In order to make such reservations clear we have now included a short section of text stating that the effect of Sec61 inhibitors on the host cell, including ACE2 biogenesis, present an obvious hurdle to their future use. We maintain that experiments in cell culture models, as performed using cycloheximide are warranted. See yellow highlighted text on page 9 of the revised manuscript.

Second decision letter

MS ID#: JOCES/2020/257758

MS TITLE: Ipomoeassin-F inhibits the in vitro biogenesis of the SARS-CoV-2 spike protein and its host cell membrane receptor

AUTHORS: Sarah O'Keefe, Peristera Roboti, Kwabena B Duah, Guanghui Zong, Hayden Schneider, Wei Q Shi, and Stephen High
ARTICLE TYPE: Short Report

Thank you for the careful revisions to your manuscript, I am happy to tell you that your manuscript has been accepted for publication in Journal of Cell Science, pending standard ethics checks.

© 2021. Published by The Company of Biologists under the terms of the Creative Commons Attribution License (https://creativecommons.org/licenses/by/4.0/).
